# Supplementary material for: Paracrine rescue of MYR1-deficient Toxoplasma gondii mutants reveals limitations of pooled in vivo CRISPR screens
Source: eLife. 2024 Dec 10;13:RP102592. doi: 10.7554/eLife.102592 (PMC11630813; doi:10.7554/eLife.102592)
Supplement: Figure 1—source data 4. [file elife-102592-fig1-data4.zip › Figure 1 - source data 4/Figure 1 - source data 4.pdf]

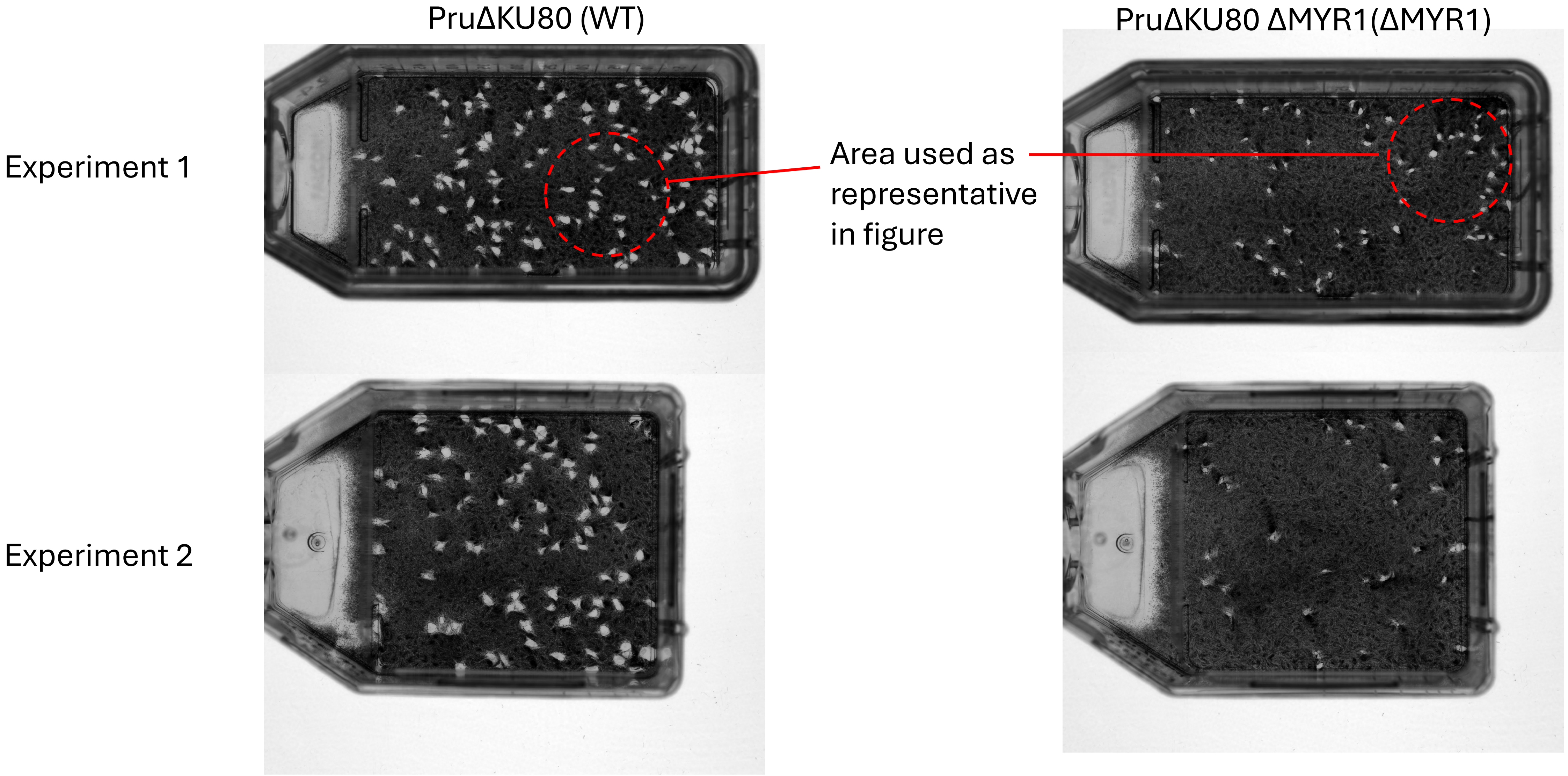

Figure 1 – source data 4. Original images of Figure 1, panel D. The plaque area of parental PruΔKU80 and derived PruΔMYR1 strains was measured via FIJI and compared.
