# Supplementary figures and images for: Paracrine rescue of MYR1-deficient Toxoplasma gondii mutants reveals limitations of pooled in vivo CRISPR screens
Source: eLife. 2024 Dec 10;13:RP102592. doi: 10.7554/eLife.102592 (PMC11630813; doi:10.7554/eLife.102592)

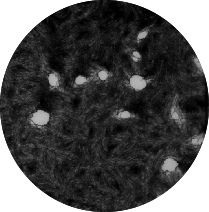

Supplement: Figure 1—source data 5. [file elife-102592-fig1-data5.zip › Figure 1 - source data 5/dfft01 myr mch(Silver Stain) copy.png]

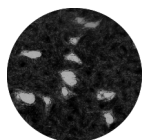

Supplement: Figure 1—source data 5. [file elife-102592-fig1-data5.zip › Figure 1 - source data 5/WT.pdf]

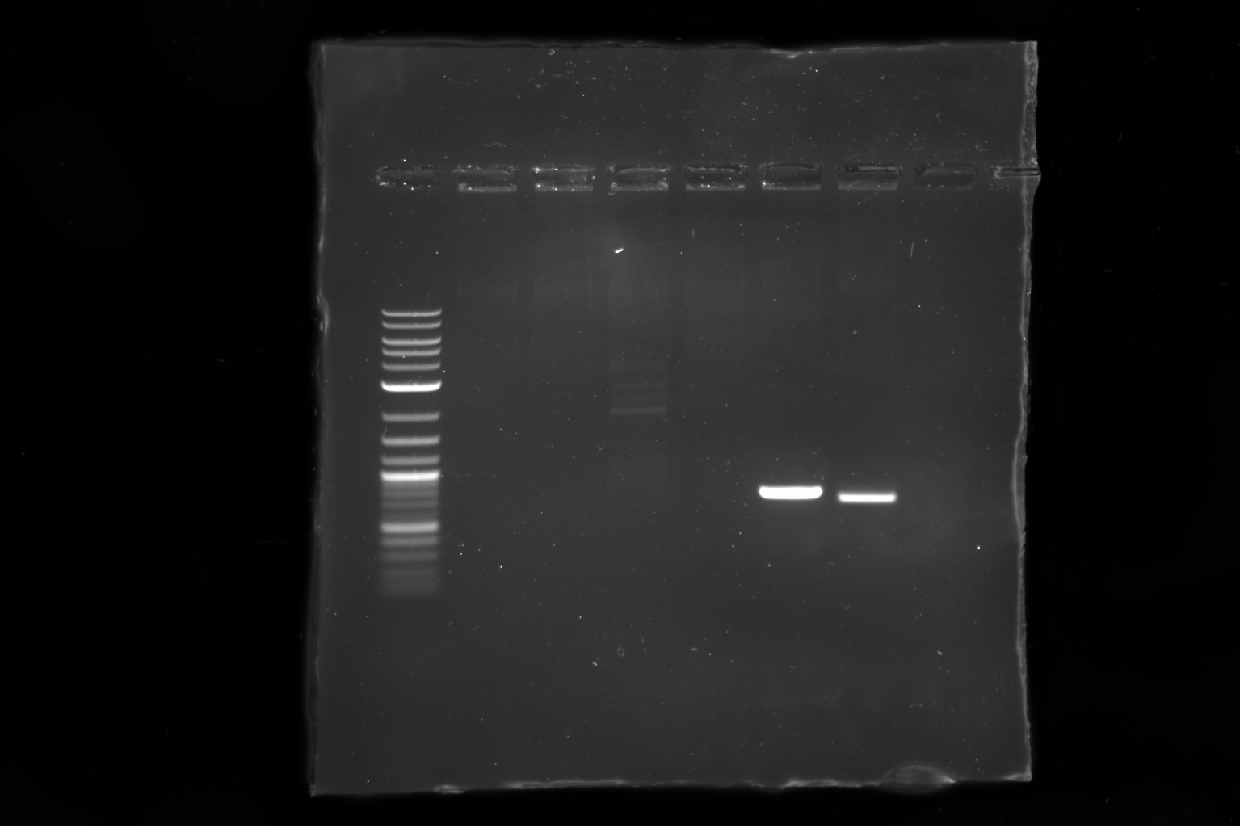

Supplement: Figure 1—figure supplement 1—source data 2. [file elife-102592-fig1-figsupp1-data2.zip › Figure 1 - Figure Supplement 1 - source data 2/igcuser 2024-07-30 18h23m12s(SYBR┬« Safe).tif]

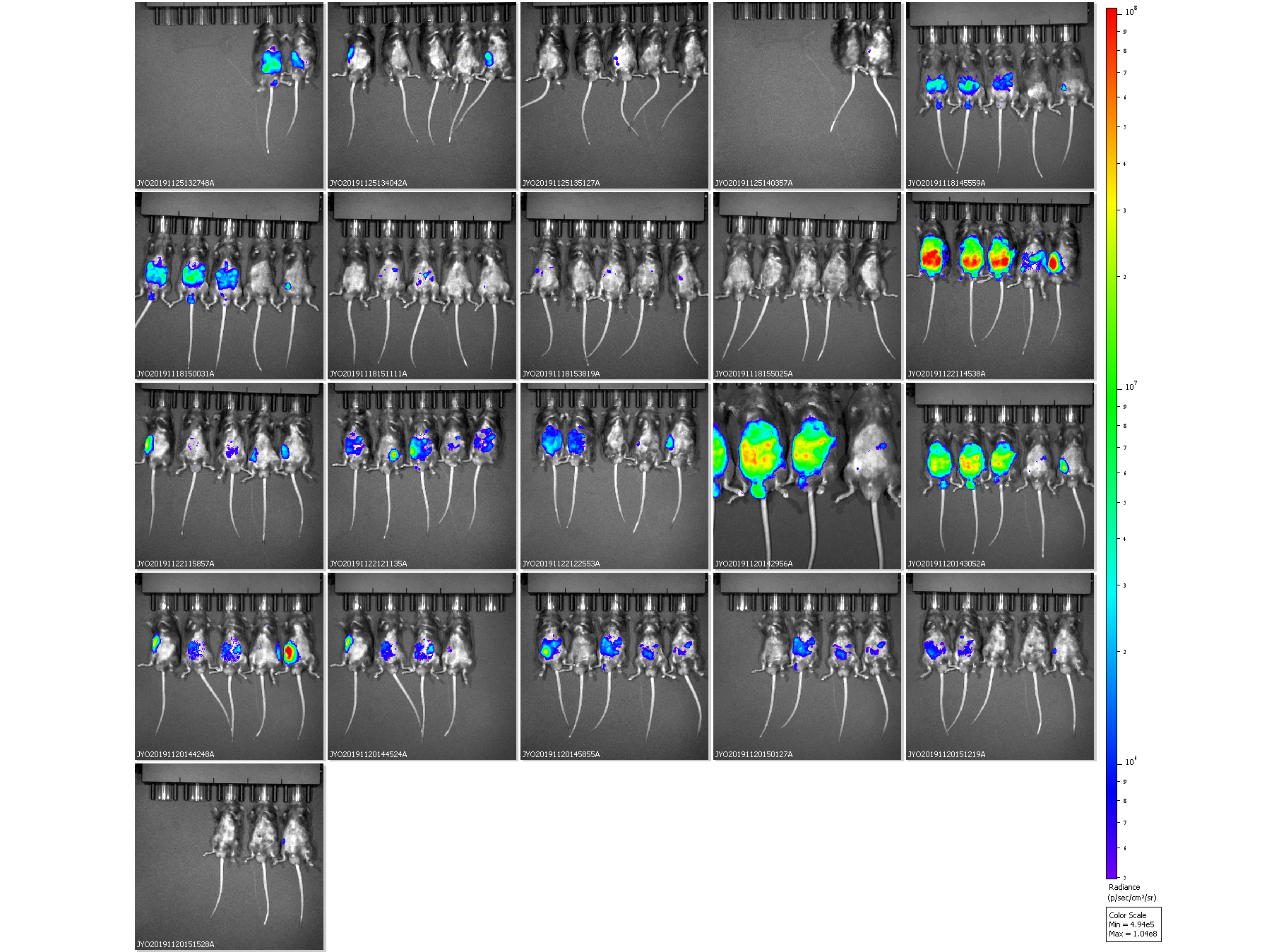

Supplement: Figure 2—source data 2. [file elife-102592-fig2-data2.zip › Figure 2 - source data 2/JYFT_151119_ALL_SCALE.png]

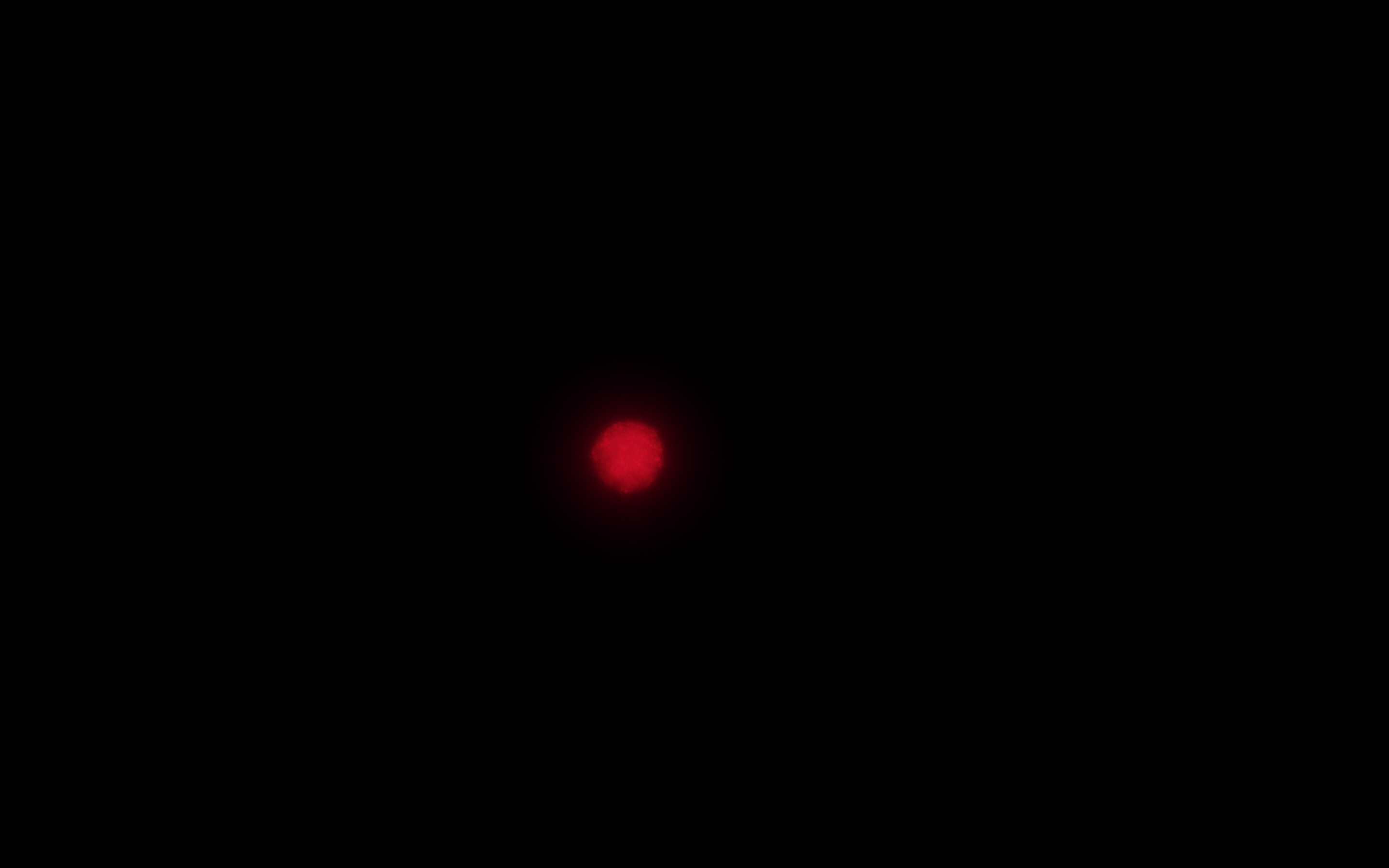

Supplement: Figure 2—source data 5. [file elife-102592-fig2-data5.zip › Figure 2 - source data 5/FT_200110_NM_cyst1_B_40x.tif]

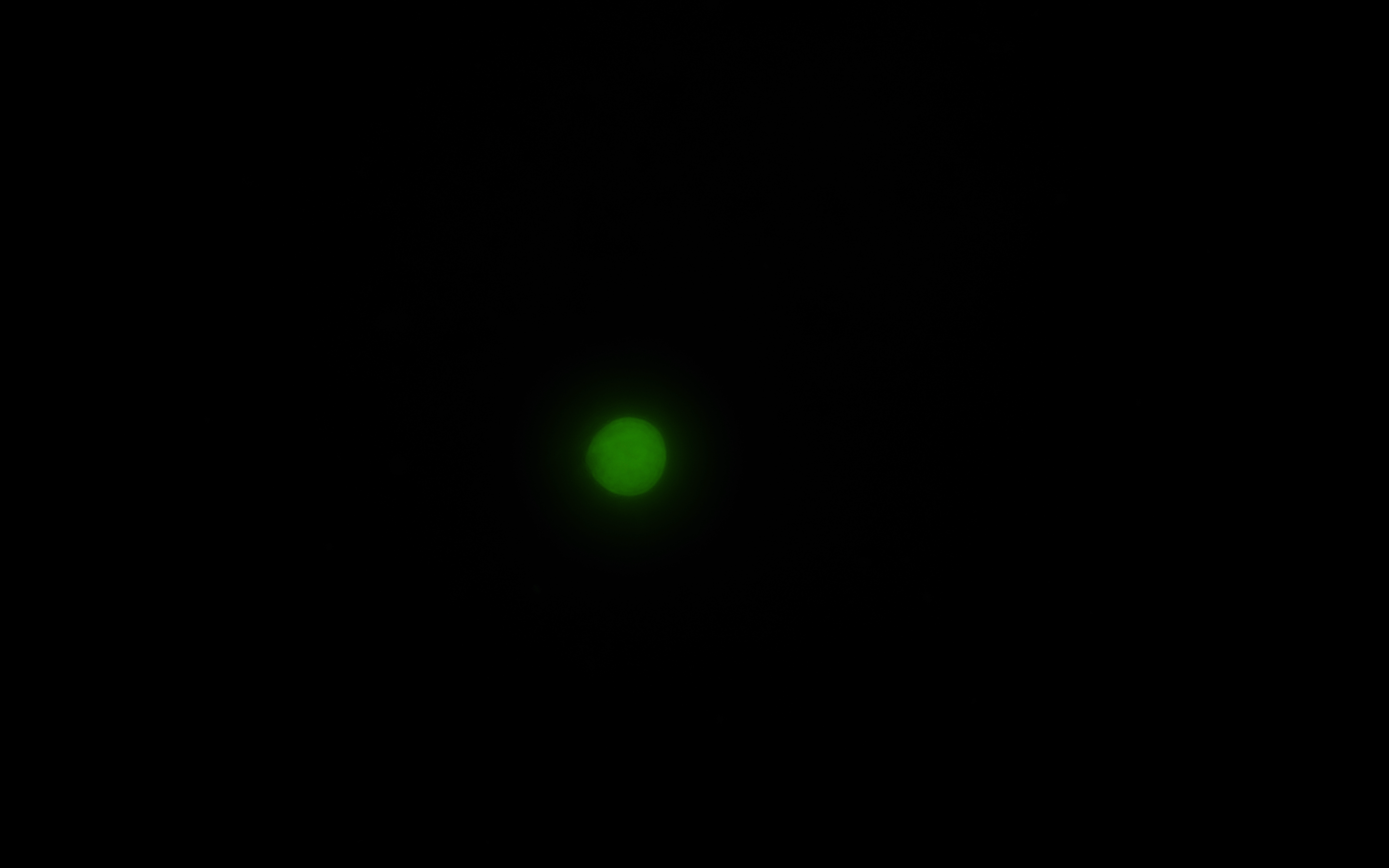

Supplement: Figure 2—source data 5. [file elife-102592-fig2-data5.zip › Figure 2 - source data 5/FT_200110_NM_cyst1_A.tif]

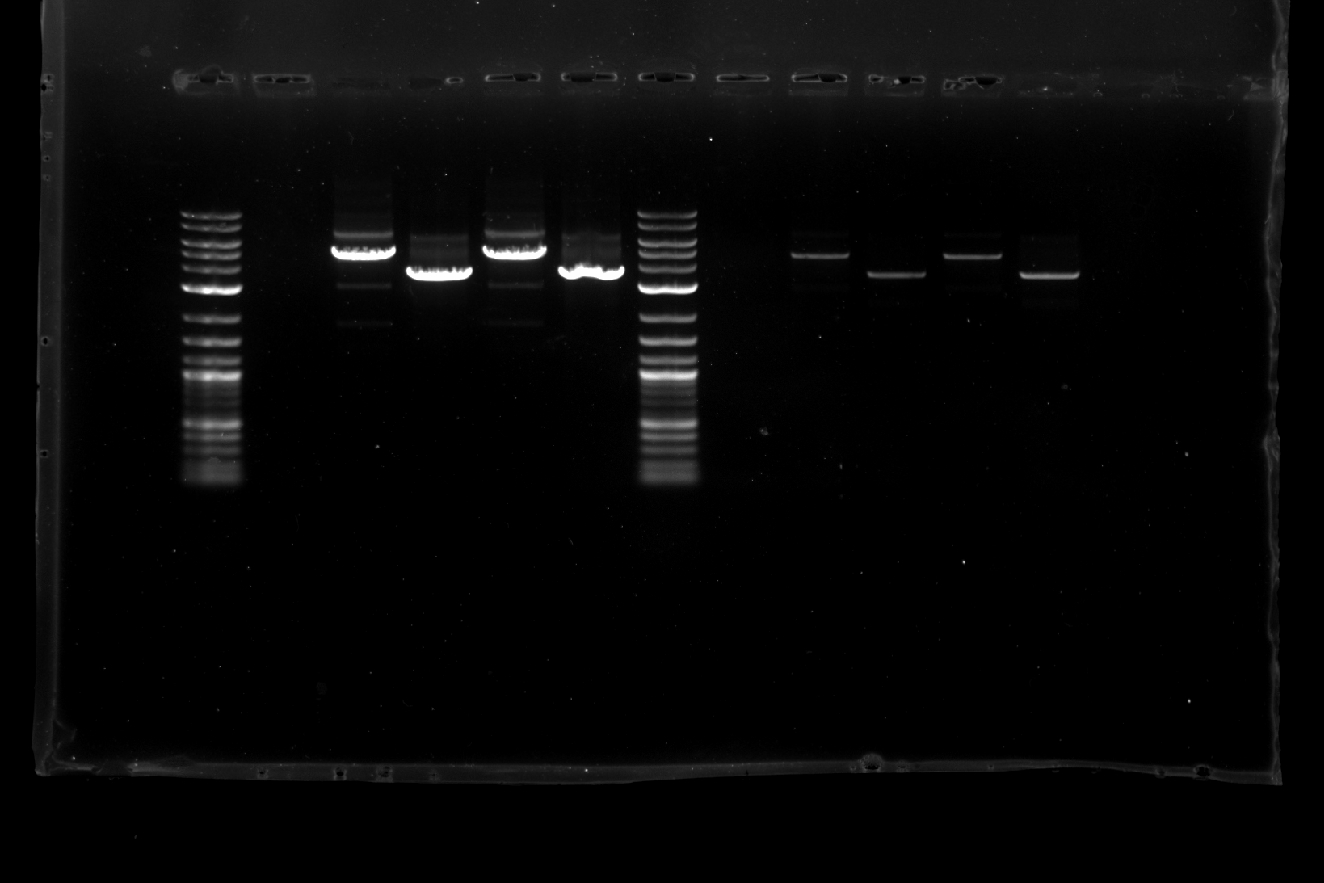

Supplement: Figure 2—figure supplement 1—source data 2. [file elife-102592-fig2-figsupp1-data2.zip › Figure 2 - Figure Supplement 2 - source data 2/igcuser 2024-08-21 10h29m23s(SYBR┬« Safe).jpg]

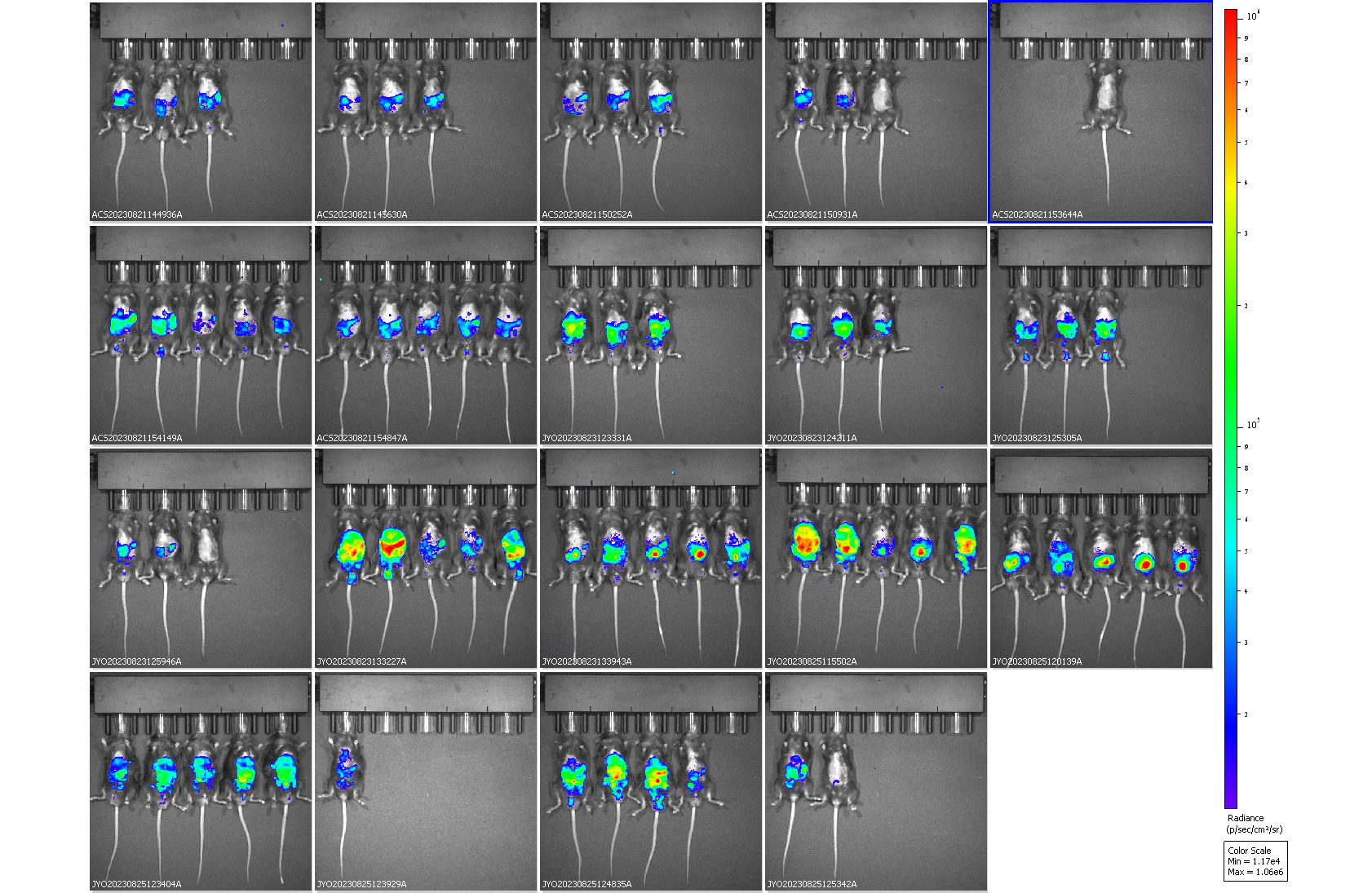

Supplement: Figure 3—source data 2. [file elife-102592-fig3-data2.zip › Figure 3 - source data 2/DFFT-03_CCR2KO_ALL_SCALE.png]

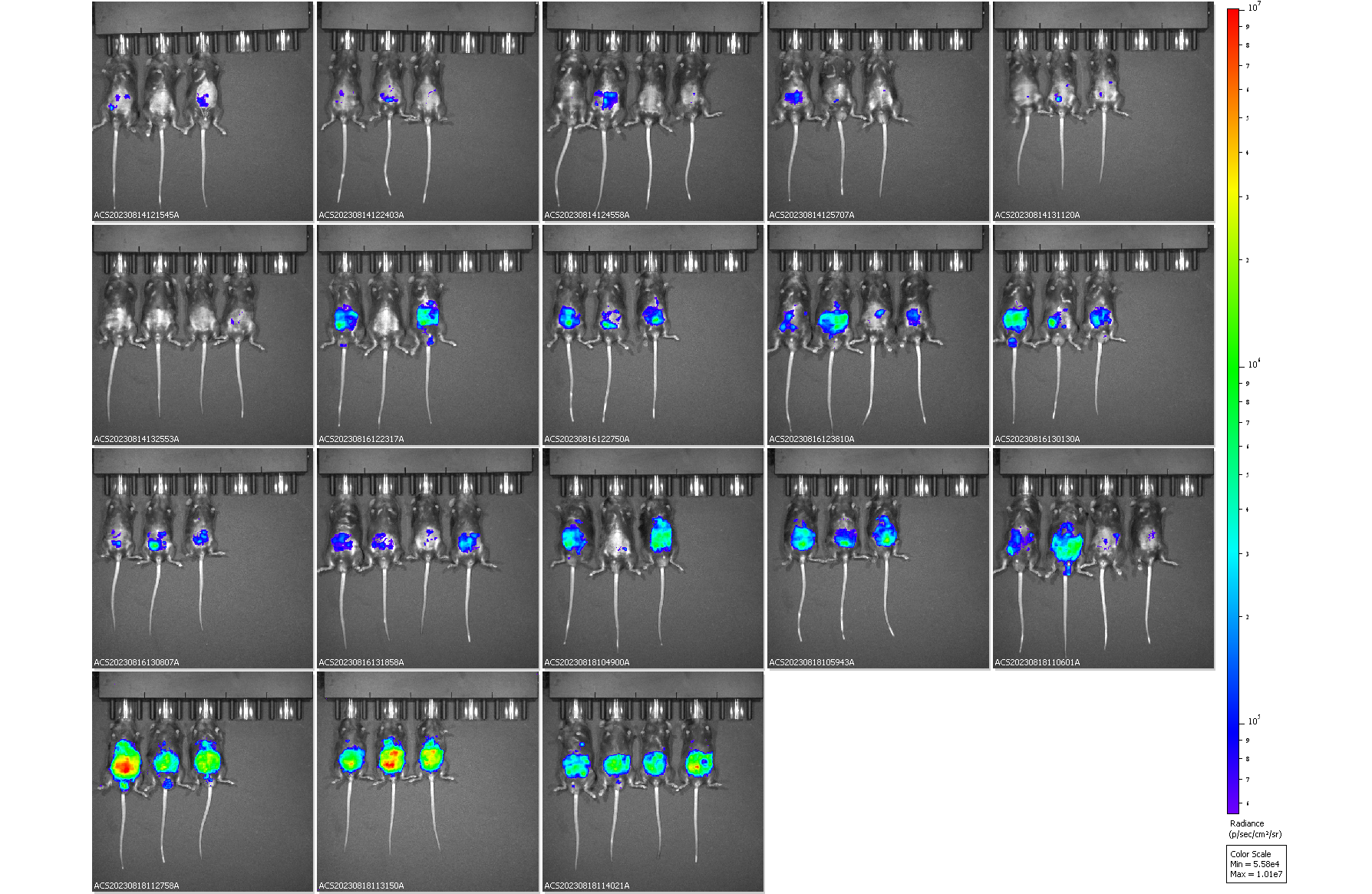

Supplement: Figure 3—source data 6. [file elife-102592-fig3-data6.zip › Figure 3 - source data 6/DFFT-01_RAG2KO_ALL_SCALE.png]
