# Supplementary material for: Paracrine rescue of MYR1-deficient Toxoplasma gondii mutants reveals limitations of pooled in vivo CRISPR screens
Source: eLife. 2024 Dec 10;13:RP102592. doi: 10.7554/eLife.102592 (PMC11630813; doi:10.7554/eLife.102592)
Supplement: Figure 1—figure supplement 1—source data 1. [file elife-102592-fig1-figsupp1-data1.zip › Figure 1 - Figure Supplement 1 - source data 1/Figure 1 - Figure Supplement 1 - source data 1.pdf]

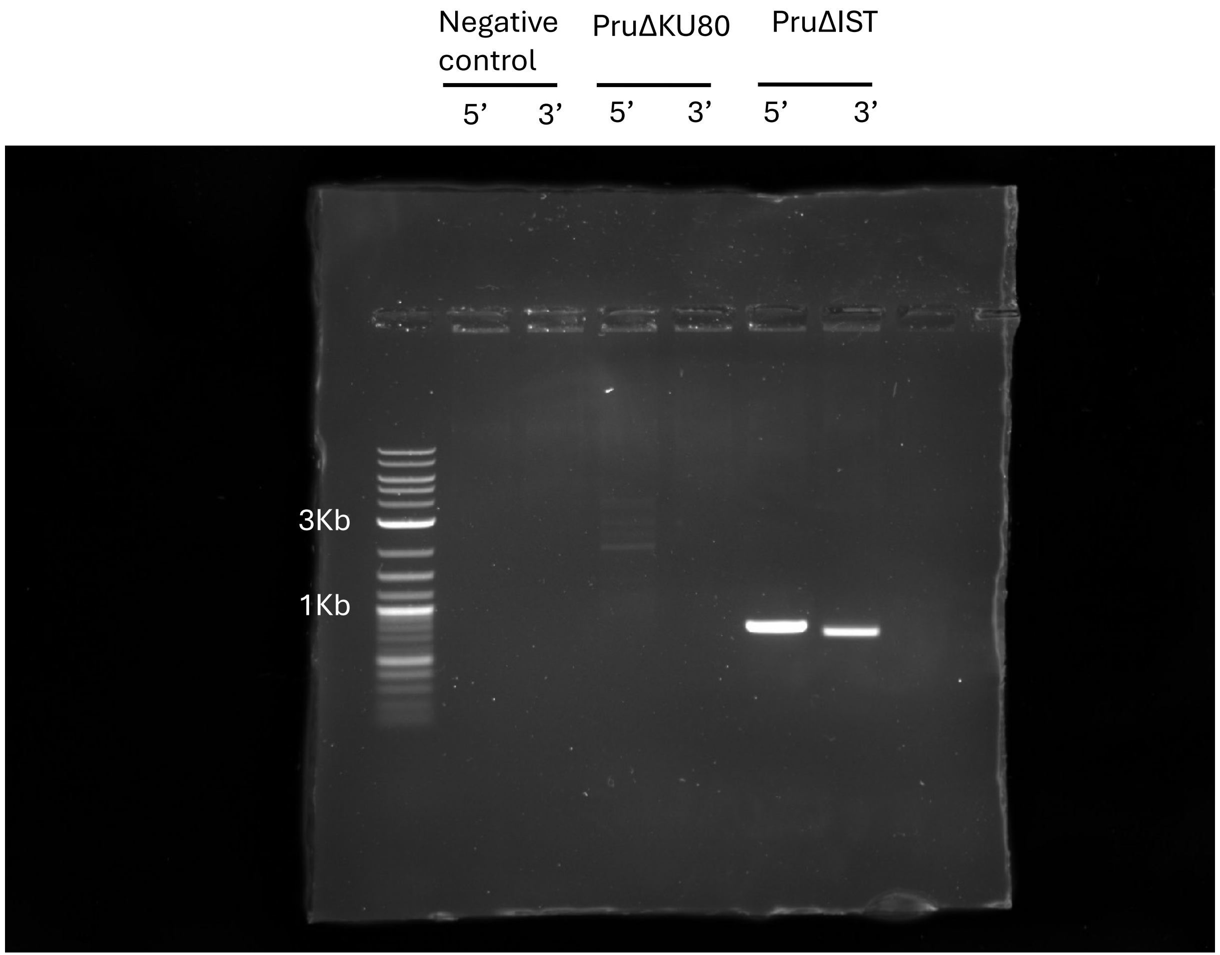

Figure 1 – Figure Supplement 1 – source data 1. PCR validation of the established PruΔIST strain compared to parental PruΔKU80 control. The 5’ and 3’ sites of the endogenous IST locus were checked for integration of the mCherry::HXGPRT repair cassette.
