# Supplementary material for: Paracrine rescue of MYR1-deficient Toxoplasma gondii mutants reveals limitations of pooled in vivo CRISPR screens
Source: eLife. 2024 Dec 10;13:RP102592. doi: 10.7554/eLife.102592 (PMC11630813; doi:10.7554/eLife.102592)
Supplement: Figure 2—source data 4. [file elife-102592-fig2-data4.zip › Figure 2 - source data 4/Figure 2 - source data 4.pdf]

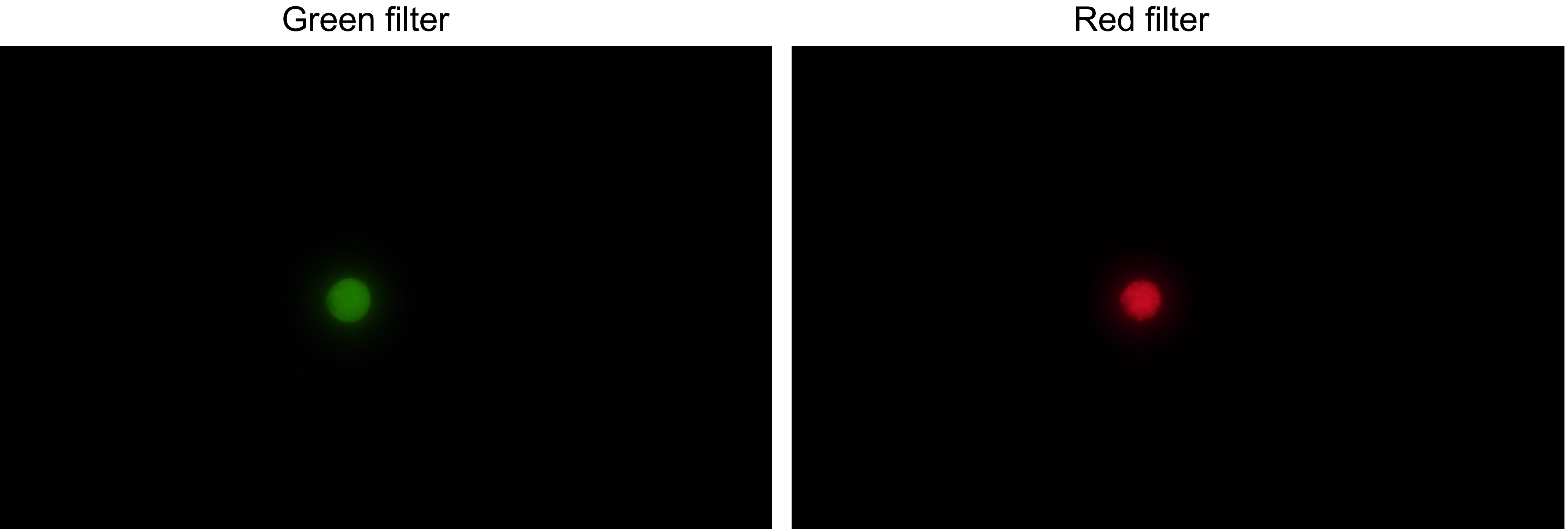

Figure 2 – source data 4. Original images of a Pru ΔMYR1 cyst (expressing constitutive mCherry) recovered from the brain of an infected C57BL/6J mouse. The brain lysate was stained with a FITC-conjugated Dolichos Biflorus Agglutinin and imaged with an inverted Ti-E Nikon microscope with LED light filters and an Olympus DF74 camera at 40x magnification.
