# Supplementary material for: Paracrine rescue of MYR1-deficient Toxoplasma gondii mutants reveals limitations of pooled in vivo CRISPR screens
Source: eLife. 2024 Dec 10;13:RP102592. doi: 10.7554/eLife.102592 (PMC11630813; doi:10.7554/eLife.102592)
Supplement: Figure 2—figure supplement 1—source data 1. [file elife-102592-fig2-figsupp1-data1.zip › Figure 2 - Figure Supplement 2 - source data 1/Figure 2 - Figure Supplement 1 - source data 1.pdf]

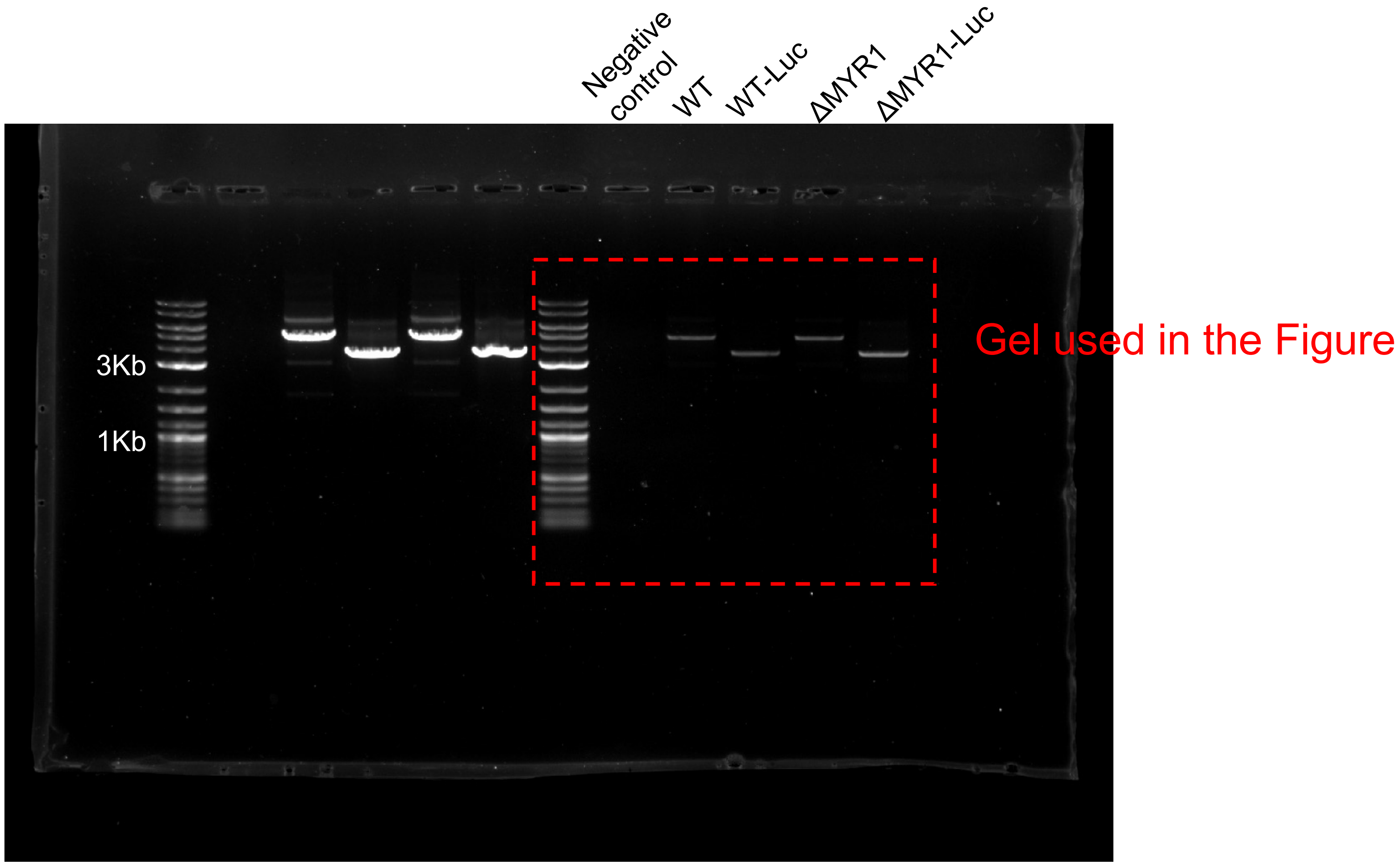

Figure 2 – Figure Supplement 1 – source data 1. PCR validation of the established PruΔKu80-LucHA (WT-Luc) and PruΔKu80ΔMYR1-LucHA (ΔMYR1-Luc) strains compared to their respective parental strains PruΔKU80 (WT) and PruΔKu80ΔMYR1 (ΔMYR1). The endogenous *Uprt* locus was checked for integration of the Luciferase gene.
