# Supplementary material for: Paracrine rescue of MYR1-deficient Toxoplasma gondii mutants reveals limitations of pooled in vivo CRISPR screens
Source: eLife. 2024 Dec 10;13:RP102592. doi: 10.7554/eLife.102592 (PMC11630813; doi:10.7554/eLife.102592)
Supplement: Figure 3—source data 1. [file elife-102592-fig3-data1.zip › Figure 3 - source data 1/Figure 3 - source data 1.pdf]

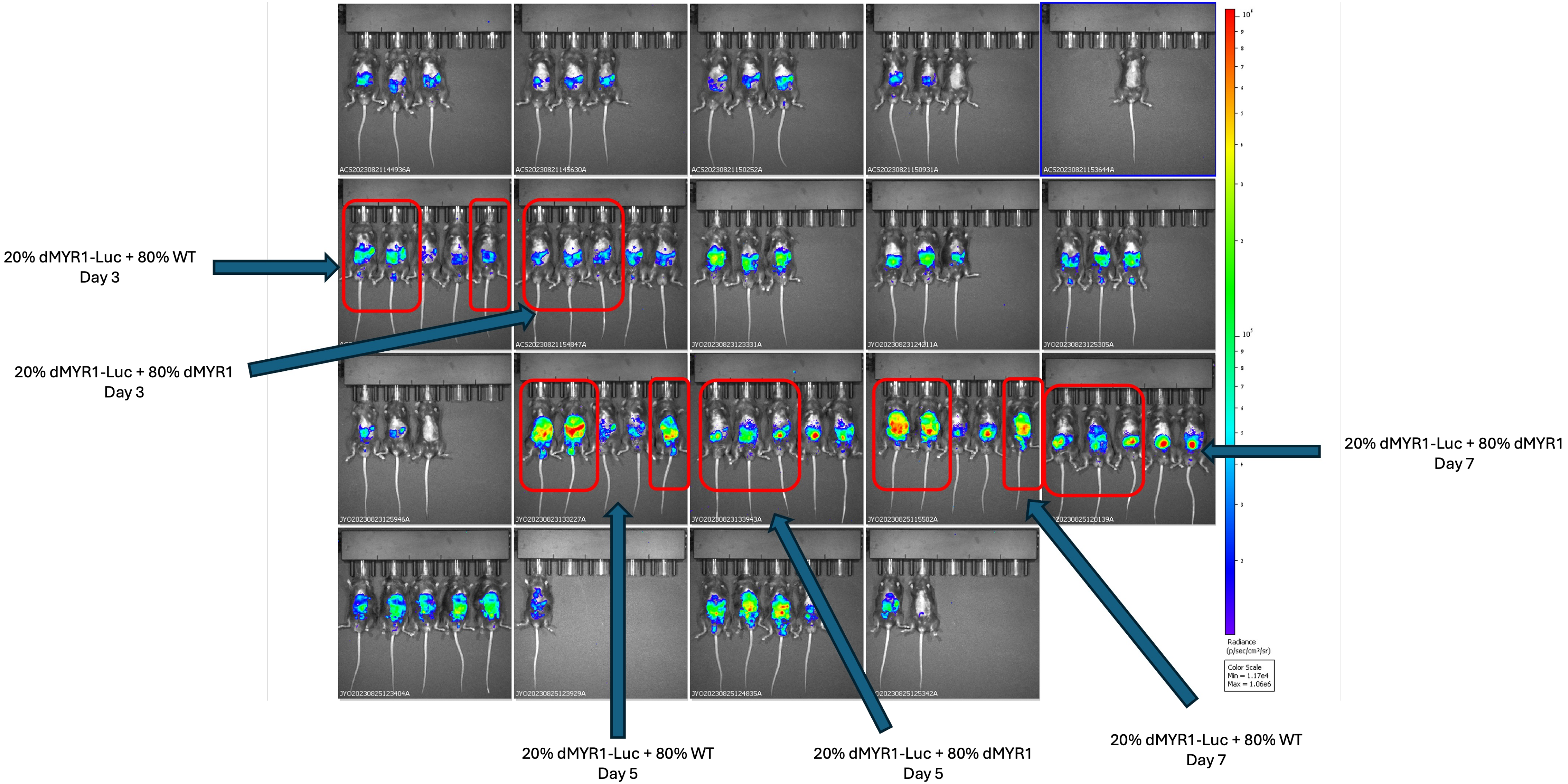

Figure 3 – source data 1. Original images of C57BL/6J mice infected with a mixed infection of 20:80 ΔMYR1-Luc:WT or ΔMYR1 parasites. ΔMYR1-Luc parasite growth was assessed via intravital imaging at day 3, 5 and 7 post infection. Highlighted the images used as representative in Figure 3, panel B.
