# Supplementary material for: Paracrine rescue of MYR1-deficient Toxoplasma gondii mutants reveals limitations of pooled in vivo CRISPR screens
Source: eLife. 2024 Dec 10;13:RP102592. doi: 10.7554/eLife.102592 (PMC11630813; doi:10.7554/eLife.102592)
Supplement: Figure 3—source data 5. [file elife-102592-fig3-data5.zip › Figure 3 - source data 5/Figure 3 - source data 5.pdf]

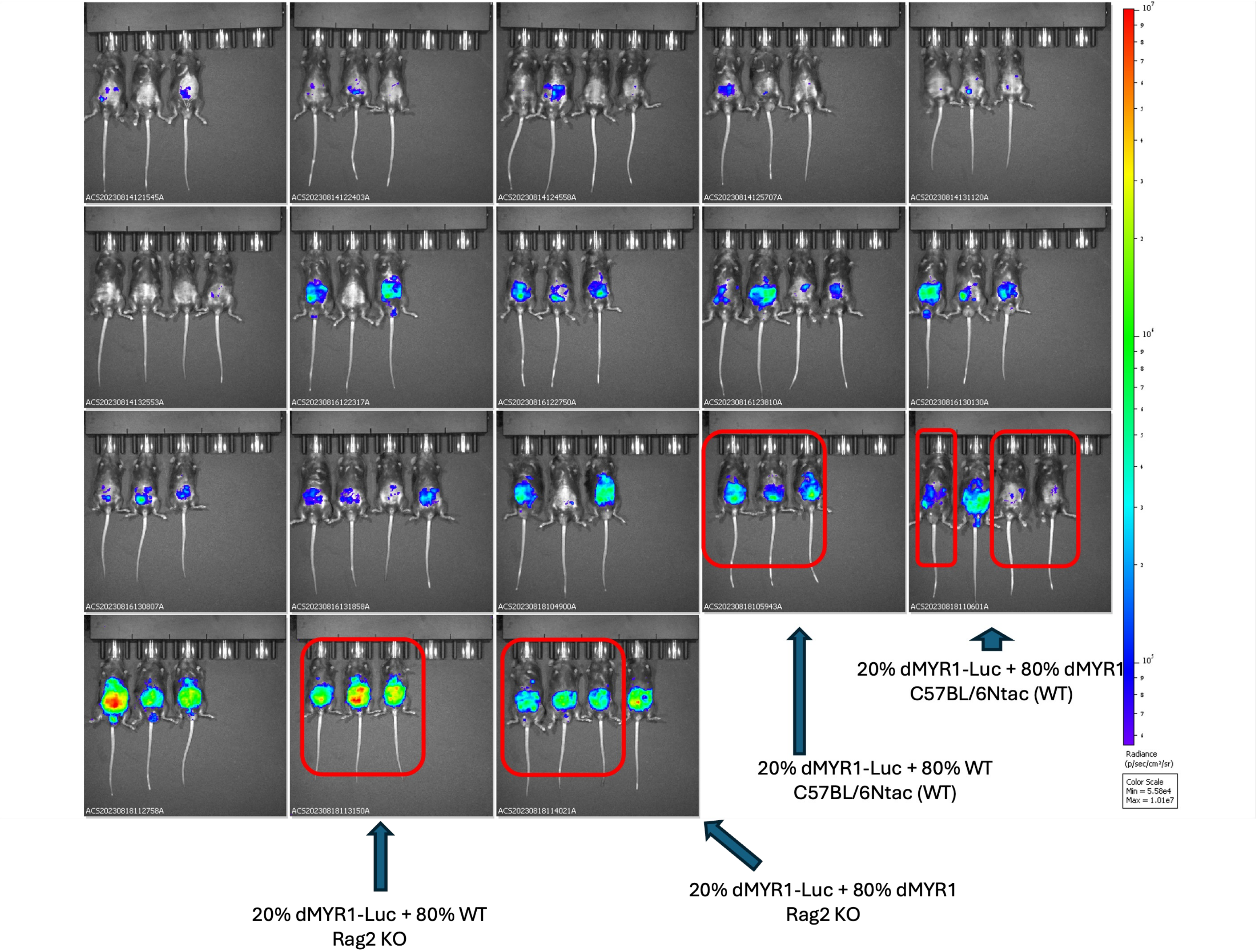

Figure 3 – source data 5. Original images of C57BL/6Ntac (WT) and RAG2 KO mice infected with a mixed infection of 20:80 ΔMYR1-Luc:WT or ΔMYR1 parasites. ΔMYR1-Luc parasite growth was assessed via intravital imaging at day 3, 5 and 7 post infection. Highlighted the images used as representative in Figure 3, panel E.
